# Progressive Enlargement of a Coronary Artery Communication With an Atrial Septal Defect Closure Device

**DOI:** 10.1016/j.jaccas.2026.108787

**Published:** 2026-06-12

**Authors:** Soichiro Kobayashi, Akihiro Takasaki, Yoshito Ogihara, Tairo Kurita, Kaoru Dohi

**Affiliations:** Department of Cardiology and Nephrology, Mie University Graduate School of Medicine, Tsu, Japan

**Keywords:** atrial septal defect, coronary angiography, secundum type

## Abstract

A 70-year-old man underwent coronary angiography 8 years after transcatheter atrial septal defect closure. Although no coronary stenosis was observed, a progressively enlarged coronary artery communication originating from the atrial branch of the left circumflex artery and extending into the device was identified. Serial imaging demonstrated progressive enlargement and elongation of this vascular structure over time. Computed tomography showed contrast opacification within the device and left-to-right leakage, suggesting incomplete neoendothelialization and peridevice leak. These findings may reflect progressive enlargement of a preexisting vascular connection associated with the device, which may interfere with endothelialization and contribute to late complications.

## Graphical Abstract

**Figure:**
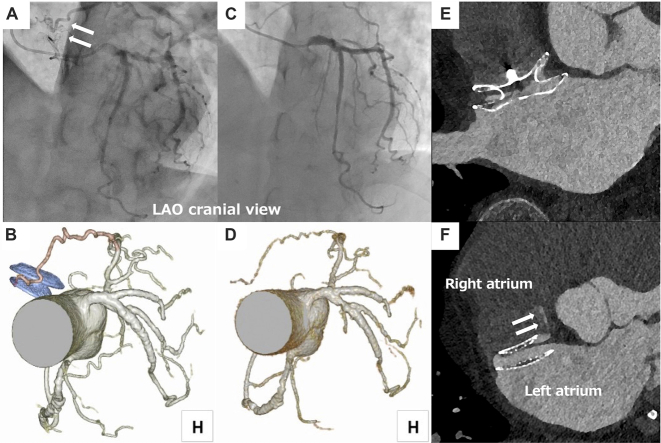

A 70-year-old man with a history of percutaneous coronary interventions for acute myocardial infarction and angina pectoris had undergone transcatheter atrial septal defect (ASD) closure using a Figulla Flex II septal occluder 8 years earlier. During the follow-up period, he subsequently underwent 2 additional percutaneous coronary interventions and was admitted for coronary angiography to re-evaluate coronary stenosis. At the time of admission, transthoracic echocardiography showed no evidence of a residual shunt or peridevice leak (PDL) surrounding the ASD closure device. Coronary angiography and computed tomography showed no stenosis but demonstrated progressive enlargement of a coronary artery communication with an ASD closure device from the atrial branch of the left circumflex artery (Figures 1A and 1B, Videos 1 and 2). This finding was not identified on previous angiography performed before ASD closure (Figures 1C and 1D, Videos 3 and 4); however, serial follow-up angiography demonstrated progressive development and elongation over time. Computed tomography demonstrated contrast opacification within the device (Figure 1E) and contrast leakage from the left to the right atrium (Figure 1F), findings that suggested incomplete neoendothelialization and PDL.

**Figure 1:**
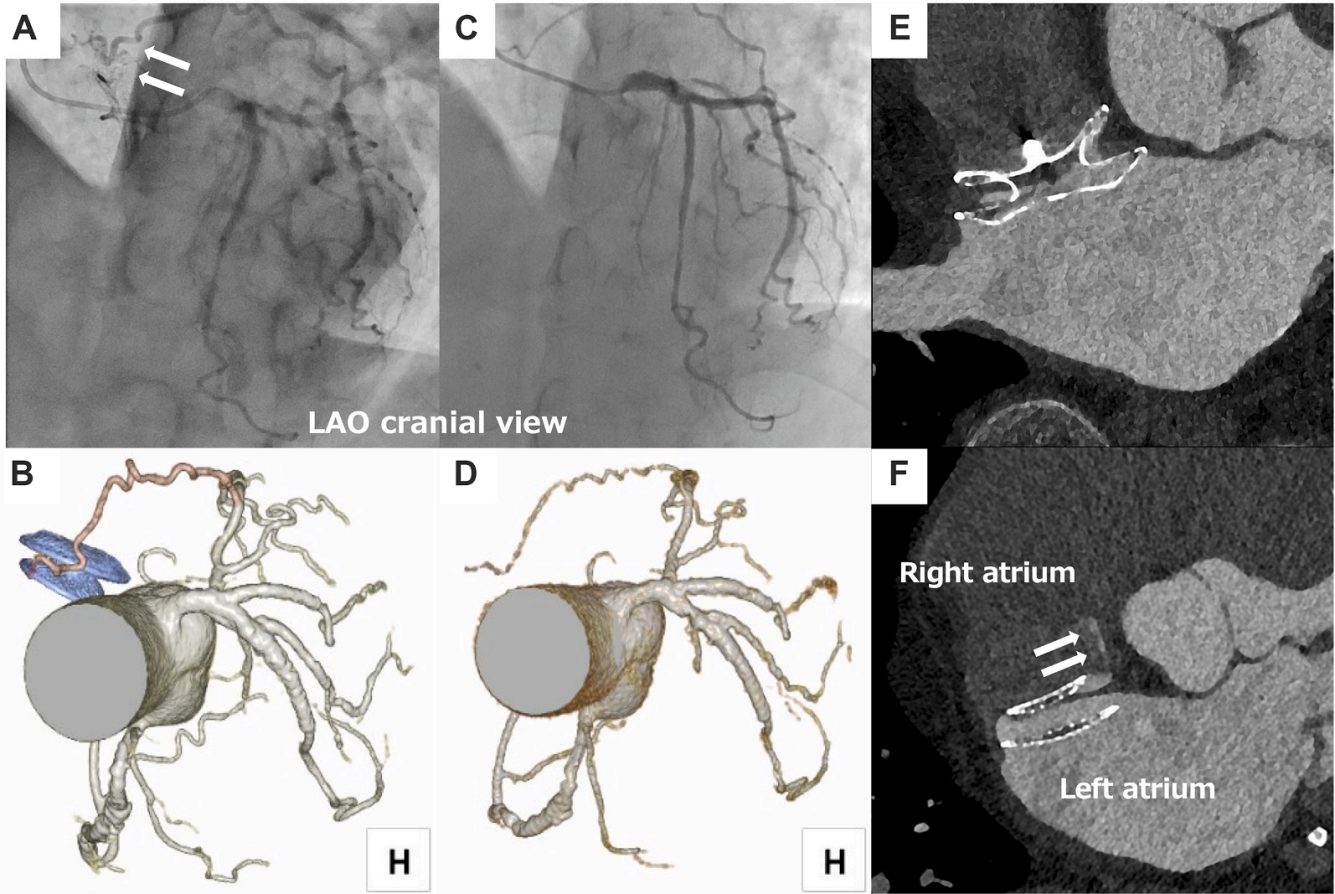
Findings on Coronary Angiography and Computed Tomography Coronary angiography (CAG) after atrial septal defect closure showed a collateral artery inside the device from the atrial branch of the left circumflex artery, highlighted by the white arrows in (A). The finding was observed on the contemporaneous computed tomography (CT) images (B), but had not been identified on previous CAG and CT images (C and D). In addition, CT showed contrast opacification within the device (E) and contrast leakage from the left to the right atrium, highlighted by white arrows in F.

To our knowledge, this is the first report demonstrating angiographic visualization of progressive enlargement of a coronary artery communication with an ASD closure device. The precise mechanism remains unclear. We speculate that long-term interaction between the device and the adjacent atrial branch, under a chronic inflammatory/healing milieu after device implantation, may have promoted progressive enlargement of a coronary artery communication with an ASD closure device.^1^^,^^2^ In turn, persistent inflow from this coronary artery communication with the device may have interfered with complete neoendothelialization and contributed to the development or persistence of PDL. This case underscores the importance of long-term multimodality imaging after ASD closure to detect unexpected device-related vascular changes.

## Funding Support and Author Disclosures

The authors have reported that they have no relationships relevant to the contents of this paper to disclose.

## Appendix

Video 1 Coronary Angiography Performed After Atrial Septal Defect Closure

Video 2 Computed Tomography Performed After Atrial Septal Defect Closure

Video 3 Coronary Angiography Performed Before Atrial Septal Defect Closure

Video 4 Computed Tomography Performed Before Atrial Septal Defect Closure
